# Colorectal cancer liver metastases – a population-based study on incidence, management and survival

**DOI:** 10.1186/s12885-017-3925-x

**Published:** 2018-01-15

**Authors:** Jennie Engstrand, Henrik Nilsson, Cecilia Strömberg, Eduard Jonas, Jacob Freedman

**Affiliations:** 1 Division of Surgery, Department of Clinical Sciences, Karolinska Institutet at Danderyd Hospital, 182 88 Stockholm, Sweden; 2 Division of Surgery, Department of Clinical Science, Intervention and Technology (CLINTEC), Karolinska Institutet at Karolinska University Hospital, 171 77 Stockholm, Sweden; 3 Surgical Gastroenterology Unit, Department of Surgery, Groote Schuur Hospital, University of Cape Town Health Sciences Faculty, Cape Town, 7925 South Africa

**Keywords:** Colorectal cancer, Liver metastases, Right-sided cancer, Left-sided cancer, Extra-hepatic metastases, Survival

## Abstract

**Background:**

Colorectal cancer (CRC) is a leading cause of cancer-associated deaths with liver metastases developing in 25–30% of those affected. Previous data suggest a survival difference between right- and left-sided liver metastatic CRC, even though left-sided cancer has a higher incidence of liver metastases. The aim of the study was to describe the liver metastatic patterns and survival as a function of the characteristics of the primary tumour and different combinations of metastatic disease.

**Methods:**

A retrospective population-based study was performed on a cohort of patients diagnosed with CRC in the region of Stockholm, Sweden during 2008. Patients were identified through the Swedish National Quality Registry for Colorectal Cancer Treatment (SCRCR) and additional information on intra- and extra-hepatic metastatic pattern and treatment were retrieved from electronic patient records. Patients were followed for 5 years or until death. Factors influencing overall survival (OS) were investigated by means of Cox regression. OS was compared using Kaplan-Meier estimations and the log-rank test.

**Results:**

Liver metastases were diagnosed in 272/1026 (26.5%) patients within five years of diagnosis of the primary. Liver and lung metastases were more often diagnosed in left-sided colon cancer compared to right-sided cancer (28.4% versus 22.1%, *p* = 0.029 and 19.7% versus 13.2%, *p* = 0.010, respectively) but the extent of liver metastases were more extensive for right-sided cancer as compared to left-sided (*p* = 0.001). Liver metastatic left-sided cancer, including rectal cancer, was associated with a 44% decreased mortality risk compared to right-sided cancer (HR = 0.56, 95% CI: 0.39–0.79) with a 5-year OS of 16.6% versus 4.3% (*p* < 0.001). In liver metastatic CRC, the presence of lung metastases did not significantly influence OS as assessed by multivariate analysis (HR = 1.11, 95% CI: 0.80–1.53).

**Conclusion:**

The worse survival in liver metastatic right-sided colon cancer could possibly be explained by the higher number of metastases, as well as more extensive segmental involvement compared with left-sided colon and rectal cancer, even though the latter had a higher incidence of liver metastases. Detailed population-based data on the metastatic pattern of CRC and survival could assist in more structured and individualized guidelines for follow-up of patients with CRC.

**Electronic supplementary material:**

The online version of this article (10.1186/s12885-017-3925-x) contains supplementary material, which is available to authorized users.

## Background

Colorectal cancer (CRC) is a leading cause of cancer-associated death in Western populations and the third most frequent cause of cancer-related death in the world [1]. Population-based studies have shown that around 25–30% of patients diagnosed with CRC develop liver metastases during the course of their disease [2, 3]. Indications for curative-intended treatment of CRC liver metastases (CRCLM) have expanded in recent years. Unfortunately, despite the oncological and surgical advances made, only about 25% of patients affected are amenable to resection, which is regarded as the only way to achieve cure [3]. Historically, the indication for resection of liver metastases was based on tumour-related factors, for example tumour number, size and distribution in the liver. Currently the focus is rather on the future liver remnant (FLR), with resectability defined as the ability to perform a complete (R0) resection, while preserving a sufficient FLR. The presence of unresectable extra-hepatic disease is still considered a contraindication to liver surgery [4]. Liver resection can achieve 5-year survival rates of above 50%, compared to only around 5% for patients treated with palliative intent [5].

Although results are not consistent, primary tumour location in terms of right- versus left-sided cancer seems to play a role in metastatic pattern and survival [6–9]. The observed differences in survival may depend on differences in embryologic origin, faecal exposure of the bowel, molecular profile, response to chemotherapy as well as the difference in time of detection with right-sided cancer generally presenting at a more advanced stage [7, 10, 11]. A number of studies on differences between right- and left-sided colon cancers that only included patients with resected stage I-III colon cancer found no difference in survival, or even improved survival for right-sided colon cancer [12, 13]. Studies on stage IV CRC showed a higher incidence of liver and lung metastases in left-sided colon cancer [6, 7]. Since right-sided metastatic cancer still implies a worse survival, it has been speculated whether the delay in diagnosis for right-sided cancer results in more extensive metastatic disease at diagnosis [7]. If so, it could explain the lower resection rate of liver metastases from right-sided cancer reported in some studies [6, 14].

The aim of the study was to describe the liver metastatic patterns and survival in a population-based cohort as a function of primary tumour characteristics and different combinations of metastatic disease.

## Methods

### Study population and data collection

Ethical approval for the study was obtained from the Regional Ethical Review Board in Stockholm who also deemed the need for informed consent unnecessary according to national regulations. All patients diagnosed with CRC in the counties of Stockholm and Gotland, Sweden from January 1st 2008 to December 31st 2008 were identified using the Swedish National Quality Registry for Colorectal Cancer Treatment (SCRCR). The register has a validated coverage of over 99% [15]. In the region, CRC is treated at 9 hospitals. Data on pre-therapy CRC staging, time and type of surgery and histopathology staging were retrieved from the registry. Patients that during the course of follow-up developed any metastases were identified by reviewing the clinical records of all patients for at least five years after time of diagnosis of the primary tumour, or until time of death. Date of diagnosis and distribution of metastases were recorded in detail. It was also noted whether patients with CRCLM were assessed by a liver multidisciplinary team (MDT), and surgical and oncological treatment were documented in detail.

### Definition of terms

Synchronously detected metastases were defined as metastases detected prior to or during resection of the primary tumour, and in the case of non-resected patients as detected prior to or concurrently with the primary tumour. The TNM stage of disease at diagnosis of the primary tumour was based on histology in resected patients and on imaging in non-resected patients. Overall survival (OS) was calculated from the date of diagnosis of the primary tumour or date of metastatic disease to the date of death, or to the date of censoring of live patients in January 2014. Perioperative deaths were included in survival analyses.

### Statistical analysis

Baseline characteristics were assessed by medians (interquartile range) for continuous variables and categorical variables were expressed as totals and frequencies. Differences in medians between groups were assessed using the Wilcoxon rank-sum test (non-normally distributed data) and the Pearson’s chi-square test was used to test differences in proportions. Logistic regression was used to calculate adjusted odds ratios (OR) and a 95% confidence interval (CI) for factors predictive of surgical resection. Variables with *p* < 0.10 in the univariate analysis were included in the multivariate model. Cox proportional hazards regression models were performed to determine factors that were associated with risk of death in the overall population, and among patients with liver metastases and presented as hazard ratio (HR) and 95% CI. Variables with *p* < 0.15 in the univariate analysis were included in the multivariate model. Survival probabilities were estimated with Kaplan-Meier plots and the log-rank test for testing equality of survival functions between groups. *P*-values <0.05 were considered significant. STATA 13 (StataCorp, College Station, Texas 77,845 USA) was used for the statistical analyses.

## Results

### Demographic and clinico-pathological features

**Fig. 1:**
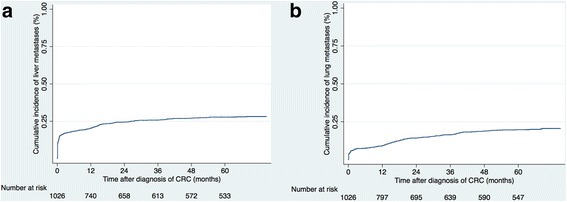
The cumulative incidence of liver metastases (**a**) and lung metastases (**b**) as related to the time of diagnosis of the primary tumour. CRC (colorectal cancer)

In 2008, a total of 1026 patients were diagnosed with CRC. During a median follow-up of 5.3 years, liver metastases were diagnosed in 272 patients (26.5%) of which 52.7% were male and 47.3% female (Table 1). In 166 patients (16.2%) the metastases were diagnosed synchronously and in 106 (10.3%) metachronously. Males were significantly younger than females at detection of the primary tumour (*p* < 0.001) but no difference in age was observed between those with or without liver metastases (*p* = 0.397). Liver metastases were detected more often in males than females (29.0% versus 23.7%, *p* = 0.054). A higher proportion of liver metastases was seen in the lower age categories as compared to the older age categories (*p* = 0.001). Liver metastases were also significantly associated with a higher T-stage and node-positive disease at diagnosis of the primary tumour. No difference was seen in the incidence of liver metastases for colon versus rectal cancer (27% versus 25%, *p* = 0.657). However, when categorizing tumours according to embryologic origin, patients with left-sided cancers (descending colon, sigmoid colon, rectum) significantly more often had liver metastases, compared to patients with right-sided cancers (cecum, ascending colon, transverse colon) (28.4% versus 22.1%, *p* = 0.029). Synchronous or metachronous detection of liver metastases was not influenced by embryologic or anatomical origin of the primary cancer (Table 1). The cumulative incidence of CRCLM as related to the time of diagnosis of the primary tumour is shown in Fig. 1a. Seventy-six percent of all liver metastases were diagnosed within the first year, and 89% and 93% within 2 and 3 years respectively.

**Table 1:** Demographic and clinico-pathological features of patients with and without liver metastases

	All patients (*n* = 1026)	No liver metastases (*n* = 754)	Liver metastases (*n* = 272)	P^a^
Age (years)^c^	71.0 (62.2–79.9)	71.9 (63.5–81.0)	68.0 (60.1–77.4)	<0.001^b^
Male	69.8 (62.1–77.5)	70.5 (62.3–78.1)	67.5 (60.3–75.1)	0.021^b^
Female	72.6 (63.3–83.2)	74.2 (65.4–84.1)	68.9 (59.4–79.9)	0.001^b^
Age category				
< 50	55 (5.4)	35 (4.6)	20 (7.4)	0.001
51–65	306 (29.8)	206 (27.3)	100 (36.8)	
66–80	413 (40.3)	309 (41.0)	104 (38.2)	
> 80	252 (24.5)	204 (27.1)	48 (17.6)	
Sex ratio (M: F)	541: 485	384:370	157: 115	0.054
Primary tumour position^d^				
Right-sided tumours	349 (34.9)	272 (36.9)	77 (29.4)	0.029
Left-sided tumours	651 (65.1)	466 (63.1)	185 (70.6)	
Primary tumour position				
Caecum/ascending colon	318 (31.0)	254 (33.7)	64 (23.5)	0.001
Transverse colon	31 (3.0)	18 (2.4)	13 (4.8)	
Descending/sigmoid colon	277 (27.0)	187 (24.8)	90 (33.1)	
Rectum	374 (36.5)	279 (37.0)	95 (34.9)	
Unknown	11 (1.0)	10 (1.3)	1 (0.4)	
Multiple primary tumours	15 (1.5)	6 (0.8)	9 (3.3)	
Tumour category^e^				
T0	12 (1.2)	11 (1.5)	1 (0.4)	
T1	90 (8.8)	85 (11.3)	5 (1.8)	<0.001
T2	145 (14.1)	138 (18.3)	7 (2.6)	
T3	520 (50.7)	389 (51.6)	131 (48.2)	
T4	201 (19.6)	105 (13.9)	96 (35.3)	
Unknown	58 (5.6)	26 (3.4)	32 (11.7)	
Node category^e^				
N0	513 (50.0)	470 (62.3)	43 (15.8)	<0.001
N1	333 (32.5)	192 (25.5)	141 (51.8)	
N2	82 (8.0)	45 (6.0)	37 (13.6)	
Unknown	98 (9.5)	47 (6.2)	51 (18.8)	
Metastatic category^e^				
M0	773 (75.4)	689 (91.4)	84 (30.9)	<0.001
M1	224 (21.8)	37 (4.9)	187 (68.8)	
Unknown	29 (2.8)	28 (3.7)	1 (0.3)	
TNM-stage^e^				
Stage I	194 (18.9)	191 (25.3)	3 (1.1)	<0.001
Stage II	299 (29.1)	274 (36.4)	25 (9.1)	
Stage III	267 (26.0)	213 (28.2)	54 (19.9)	
Stage IV	224 (21.8)	37 (4.9)	187 (68.8)	
Unknown	42 (0.4)	39 (5.2)	3 (1.1)	

Values in parentheses are percentages unless indicated otherwise

^a^Chi^2^ -test, except

^b^Wilcoxon rank-sum test

^c^Values are median (i.q.r)

^d^According to embryologic origin excluding unknown primaries (*n* = 11) and multiple primaries (*n* = 15)

^e^Stage at initial diagnosis

### Intra- and extra-hepatic metastatic pattern

At the time of diagnosis of liver metastases, 81 patients (48.8% of those with synchronous liver metastases) had liver-only metastases with 60 patients (36.1%) having widespread metastases engaging all liver segments. Twenty-two patients with metachronously detected liver metastases had liver-only metastases of which none developed any extra-hepatic metastases during follow-up. A single liver metastasis was detected in 55 patients (20.2%) and 148 patients (54.4%) had four or more tumours (Table 2). A higher tumour burden (number of metastases and number of involved segments) was seen when liver metastases were synchronously detected, as compared with metachronous detection (Table 2).

**Table 2:** Characteristics of synchronous and metachronous liver metastases

	All liver metastases (*n* = 272)	Synchronous (*n* = 166)	Metachronous (*n* = 106)	P^a^
Sex ratio (M: F)	157: 115	93: 73	64: 42	0.478
Age category				
< 50	20 (7.4)	12 (7.2)	8 (7.6)	0.950
51–65	100 (36.8)	61 (36.8)	39 (36.8)	
66–80	104 (38.2)	62 (37.4)	42 (39.6)	
> 80	48 (17.6)	31 (18.6)	17 (16.0)	
Primary tumour position^b^ (*n* = 262)				
Right-sided tumours	77 (29.4)	53 (33.1)	24 (23.5)	0.096
Left-sided tumours	185 (70.6)	107 (66.9)	78 (76.5)	
Primary tumour position^c^				
Caecum/ascending colon	64 (24.4)	42 (26.2)	22 (21.6)	0.127
Transverse colon	13 (5.0)	11 (6.9)	2 (2.0)	
Descending /sigmoid colon	90 (34.4)	56 (35.0)	34 (33.3)	
Rectum	95 (36.2)	51 (31.9)	44 (43.1)	
Number of liver metastases				
1	55 (20.2)	17 (10.2)	38 (35.8)	<0.001
2–3	69 (25.4)	37 (22.3)	32 (30.2)	
≥ 4	148 (54.4)	112 (67.5)	36 (34.0)	
No of segments involved				
1–3	138 (50.7)	64 (38.5)	74 (69.1)	<0.001
4–6	54 (19.9)	36 (21.7)	18 (17.0)	
7–8	80 (29.4)	66 (39.8)	14 (13.2)	

Values in parentheses are percentages unless indicated otherwise

^a^Chi^2^-test

^b^According to embryologic origin excluding unknown primaries (*n* = 1) and multiple primaries (*n* = 9)

^c^Excluding unknown primaries (*n* = 1) and multiple primaries (*n* = 9)

Extra-hepatic metastases were detected in 251 patients (24.5%). The lungs were the most common extra-hepatic metastatic site (174 patients, 16.9%), followed by peritoneal metastases (73 patients, 7.1%) and distant lymph node metastases (49 patients, 4.8%). Within the first year after diagnosis of CRC, the cumulative incidence of lung metastases was 51% and at 3 years, 84% of all lung metastases were diagnosed (Fig. 1b). Patients with metachronously detected liver metastases were significantly more often diagnosed with lung metastases (56.6% versus 44.0%, *p* = 0.042). Lung metastases were more frequent in left-sided colon and rectal cancer, (19.7% versus 13.2%, *p* = 0.010), and peritoneal metastases were more frequent in right-sided colon cancer (10.6% versus 5.5%, *p* = 0.003).

Liver metastatic patterns in patients with right-sided versus left-sided colon and rectal cancer are shown in Table 3. Patients with right-sided colon cancer and liver metastases had a higher tumour burden in terms of number of metastases and the number of segments involved, compared to patients with liver metastases originating from left-sided colon and rectal cancer.

**Table 3:** Characteristics of liver metastatic patterns in patients with right-sided vs. left-sided tumours

	All liver metastases^a^	Right-sided tumours^a^	Left-sided tumours^a,b^	P^c^
Total	262	77 (29.4)	185 (70.6)	0.029
Age category				
< 50	20 (7.6)	7 (9.1)	13 (7.0)	0.462
1–65	97 (37.0)	23 (29.9)	74 (40.0)	
66–80	99 (37.8)	33 (42.8)	66 (35.7)	
> 80	46 (17.6)	14 (18.2)	32 (17.3)	
Sex ratio (M: F)	150: 112	41: 36	109: 76	0.398
TNM stage at initial diagnosis^d^				
I	3 (1.2)	0 (0.0)	3 (1.7)	0.017
II	25 (9.6)	2 (2.6)	23 (12.6)	
III	51 (19.7)	12 (15.6)	39 (21.4)	
IV	180 (69.5)	63 (81.8)	117 (64.3)	
Number of liver metastases				
1	54 (20.6)	5 (6.5)	49 (26.5)	0.001
2–3	65 (24.8)	20 (26.0)	45 (24.3)	
≥ 4	143 (54.6)	52 (67.5)	91 (49.2)	
Number of segments involved				
1–3	133 (50.8)	29 (37.6)	104 (56.2)	0.019
4–6	53 (20.2)	18 (23.4)	35 (18.9)	
7–8	76 (29.0)	30 (39.0)	46 (24.9)	

Values in parentheses are percentages unless indicated otherwise

^a^Excluding unknown primaries (*n* = 1) and multiple primaries (*n* = 9)

^b^Including rectal cancer

^c^Chi^2^-test

^d^Excluding unknown TNM stage (*n* = 3)

### Treatment of liver metastases

A total of 102 patients (37.5%) were referred to a liver MDT conference and 69 of the 272 patients (25.4%) were treated with curative intent. No patients treated outside of a liver MDT conference had a liver resection. Recurrence of liver metastases was diagnosed in 29 patients, corresponding to a recurrence rate of 42%. Of these, 11 patients (38%) were re-resected. Patients with metachronous detection of liver metastases were more likely to undergo an intervention with curative intent than patients with synchronously detected metastases (33% versus 16.9%, *p* = 0.002) and major resections were less likely to be performed in the latter group (*p* = 0.001). Patients with liver metastatic left-sided cancer were more often resected, compared to patients with liver metastatic right-sided cancer (30.8% versus 14.2%, *p* = 0.005). In a multivariate logistic regression, the probability of undergoing a liver resection was associated with age ≤ 68 years (OR = 2.71, 95% CI: 1.29–5.69), primary tumour-stage T-stage (T3-T4 versus T1-T2, OR = 0.16, 95% CI: 0.03–0.87) and number of liver metastases (>5 versus 1–2, OR = 0.07, 95% CI: 0.03–0.19) (Table 4), while gender (OR = 0.94, 95% CI: 0.45–1.98), nodal stage of the primary (N0 versus N+, OR = 0.72, 95% CI: 0.31–1.67), synchronous versus metachronous detection (OR = 1.20, 95% CI:0.57–2.55) and primary tumour origin (right-sided versus left-sided, OR = 1.92, 95% CI: 0.81–4.52) were not. Thirty-nine patients (56%) in whom liver metastases were resected received pre-operative chemotherapy. There was no statistically significant difference in administration of palliative chemotherapy or best supportive care (no chemotherapy) between synchronous or metachronous detected liver metastases (*p* = 0.521). Of the 251 patients with extra-hepatic metastases, 30 (12%) were treated with curative intent (22 with surgical resection and 8 with stereotactic radiotherapy).

**Table 4:** Univariate and multivariate logistic regression analysis of patient and tumour factors associated with the probability of undergoing a liver intervention for CRCLM

	Univariate analysis^a^		Multivariate analysis^a^	
	Odds ratio (95% CI)	*P*	Odds ratio (95% CI)	*P*
Patient factors				
Age (years)				
> 68	1.00 (reference)		1.00 (reference)	
≤ 68	2.93 (1.63–5.24)	<0.001	2.71 (1.29–5.69)	0.009
Sex				
Female	1.00 (reference)		1.00 (reference)	
Male	1.52 (0.86–2.69)	0.146	0.94 (0.45–1.98)	0.879
Tumour factors				
Tumour stage^b^				
T1 or T2	1.00 (reference)		1.00 (reference)	
T3 or T4	0.31 (0.10–0.97)	0.045	0.16 (0.03–0.87)	0.035
Nodal stage^b^				
N0	1.00 (reference)		1.00 (reference)	
N1 or N2	0.31 (0.16–0.62)	0.001	0.72 (0.31–1.67)	0.440
Time of detection				
Metachronous	1.00 (reference)		1.00 (reference)	
Synchronous	0.41 (0.24–0.72)	0.002	1.20 (0.57–2.55)	0.626
No of liver metastases				
1–2	1.00 (reference)		1.00 (reference)	
3–4	0.70 (0.32–1.56)	0.387	0.90 (0.33–2.46)	0.844
≥ 5	0.06 (0.02–0.14)	<0.001	0.07 (0.03–0.19)	<0.001
Tumour origin				
Right-sided	1.00 (reference)		1.00 (reference)	
Left-sided^c^	2.67 (1.31–5.44)	0.007	1.92 (0.81–4.52)	0.136

Values in parentheses are 95% CI

^a^Logistic regression with liver intervention as dependent variable

^b^Stage at initial diagnosis

^c^Including rectal cancer

### Survival

Five-year OS for the entire CRC cohort was 56.2%. Factors influencing OS among patients with liver metastases are shown in Table 5. In the multivariate Cox regression analysis, primary tumour location was a significant prognostic factor for survival with better survival for left-sided colon and rectal cancer (HR = 0.56, 95% CI: 0.39–0.79). The other factors that remained significant in the multivariate analysis were age (HR = 1.03, 95% CI: 1.01–1.05), size of liver metastases >50 mm (HR = 2.51, 95% CI 1.73–3.65) and liver resection (HR = 0.21, 95% CI: 0.13–0.33) (Table 5). In the stage-adjusted multivariate analysis, primary tumour site in liver metastatic cancer remained a prognostic factor for survival in stage III (HR = 0.13, 95% CI: 0.05–0.35) and stage IV CRC (HR = 0.65, 95% CI: 0.47–0.90) while stage II (HR = 6.40, 95% CI: 0.62–66.00) did not (Additional file 1: Table S1). The limited number of patients with stage I disease, and to some extent stage II disease, resulted in hazard ratio estimations with extremely wide CI. When excluding rectal cancer from left-sided colon cancer, site of primary tumour remained significant in the multivariate analysis among patients with liver metastases (HR = 0.56, 95% CI: 0.36–0.86) and non-significant (HR = 1.02, 95% CI: 0.81–1.28) in the univariable analysis of the overall population, adjusting for the same factors as in Table 5. In liver metastatic CRC, the presence of lung metastases did not significantly influence OS as assessed by multivariate analysis (HR = 1.11, 95% CI: 0.80–1.53) (Table 5).

**Table 5:** Univariate and multivariate Cox regression analysis of factors influencing OS in patients with CRCLM

	Univariate analysis		Multivariate analysis	
	Hazard ratio	*P*	Hazard ratio	*P*
Age (years)^a^	1.03 (1.02–1.05)	<0.001	1.03 (1.01–1.05)	0.002
Sex				
Female	1.00 (reference)		1.00 (reference)	
Male	0.80 (0.62–1.04)	0.099	0.91 (0.65–1.28)	0.598
Tumour stage^b^				
T1-T2	1.00 (reference)			
T3-T4	1.03 (0.56–1.89)	0.929		
Nodal stage^c^				
N0	1.00 (reference)		1.00 (reference)	
N1-N2	1.65 (1.10–2.46)	0.014	0.89 (0.57–1.37)	0.586
Primary tumour origin				
Right-sided	1.00 (reference)		1.00 (reference)	
Left-sided^d^	0.51 (0.39–0.68)	<0.001	0.56 (0.39–0.79)	0.001
Time of detection of liver metastases				
Metachronous	1.00 (reference)		1.00 (reference)	
Synchronous	1.49 (1.14–1.94)	0.004	0.91(0.64–1.30)	0.606
Size of largest liver metastasis (mm)				
< 50 mm	1.00 (reference)		1.00 (reference)	
> 50 mm	3.07 (2.34–4.03)	<0.001	2.51 (1.73–3.65)	<0.001
Number of liver metastases				
1–2	1.00 (reference)		1.00 (reference)	
3–4	1.06 (0.67–1.68)	0.800	1.06 (0.60–1.89)	0.836
≥ 5	2.83 (2.12–3.77)	0.000	1.41 (0.94–2.11)	0.095
Liver resection				
No	1.00 (reference)		1.00 (reference)	
Yes	0.14 (0.10–0.21)	<0.001	0.21 (0.13–0.33)	<0.001
Lung metastases				
No	1.00 (reference)		1.00 (reference)	
Yes	1.26 (0.98–1.64)	0.073	1.11 (0.80–1.53)	0.528

Values in parentheses are 95% CI. OS from date of diagnosis of liver metastases

^a^Continuous variable

^b^Excluding unknown T-stage (*n* = 32)

^c^Excluding unknown nodal stage (*n* = 31)

^d^Including rectal cancer

The proportional hazards function was tested graphically and was found to be valid.

**Fig. 2:**
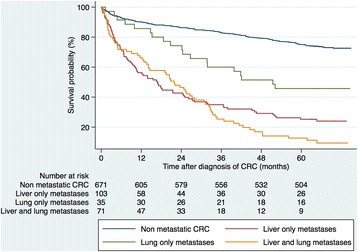
Kaplan-Meier estimates showing overall survival in patients with non-metastatic CRC and different metastatic pattern. Liver and lung metastases versus liver-only metastases, median survival 1.8 years and 1.4 years, respectively, *p* = 0.204 (log rank test). Liver-only metastases versus lung only metastases, median survival 1.4 years and 4.3 years, respectively, *p* = 0.006 (log-rank test). Lung only metastases versus non-metastatic CRC *p* < 0.001, (log-rank test). CRC (colorectal cancer)

As could be expected, patients with liver metastases had a significantly lower 5-year OS compared to patients without liver metastases (16.9% versus 70.4%, *p* = 0.001). The survival of patients with non-metastatic CRC patients and liver-only metastases, lung only metastases, and liver and lung metastases combined are shown in Fig. 2. Patients without any metastatic disease had a 5-year OS of 75.1% compared to 25.2%, 45.7% and 12.7% respectively for patients with liver-only metastases, lung only metastases, and liver and lung metastases combined.

**Fig. 3:**
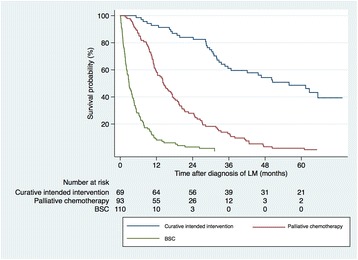
Kaplan-Meier estimates showing overall survival in patients with liver metastases treated with curative intended intervention, palliative chemotherapy or BSC (no chemotherapy). BSC versus palliative chemotherapy (median survival 0.24 years versus 1.2 years) *p* < 0.001 (log-rank test), palliative chemotherapy versus curative intended interventions (median survival 1.2 years versus 4.7 years), *p* < 0.001 (log-rank test). BSC (best supportive care)

The 1- and 5-year survival of patients with liver metastases treated with resection was 92.8% and 48.6% respectively. Patients treated with palliative chemotherapy had a 1- and 5-year survival of 58.1% and 2.2% respectively, while patients receiving best supportive care (BSC) had a 1-year survival of 8.2% and there were no 5-year survivors (Fig. 3).

**Fig. 4:**
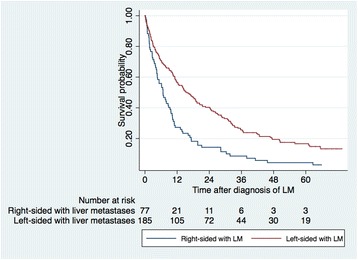
Kaplan-Meier estimates showing overall survival in patients with liver metastatic right-sided and left-sided colon and rectal cancer. Left-sided versus right-sided cancer with liver metastases (median survival 17.7 versus 6.7 months) (*p* < 0.001) (log-rank test). LM (liver metastases)

**Fig. 5:**
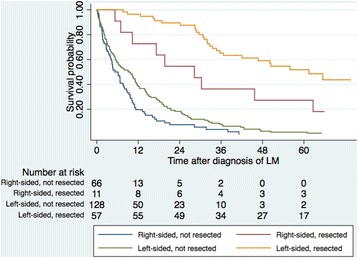
Kaplan-Meier estimates showing overall survival in patients with right-sided or left-sided colon and rectal cancer undergoing liver resection or not. Resected liver metastatic left-sided versus right-sided cancer (*p* = 0.012). Non-resected liver metastatic left-sided versus right-sided cancer (*p* = 0.007). LM (liver metastases)

In patients with liver metastases, right-sided cancers had a significantly worse OS compared to left-sided colon and rectal cancers with 2-year survival rates of 14.3% and 40.3%, respectively, and 5-year survival rates of 4.3% and 16.6%, respectively (*p* < 0.001), irrespective of treatment strategy and counting from date of diagnosis of liver metastases (Fig. 4). When taking treatment into account, patients with resected liver metastases from left-sided cancers had a 5-year survival of 51.8% while patients resected for their liver metastases originating from a right-sided tumour had a 5-year survival of 27.3% (*p* = 0.012) (Fig. 5). There was also a significant survival difference between right-sided and left-sided liver metastatic cancer if not resected (*p* = 0.007) (Fig. 5). The same survival pattern, with superior survival in left-sided cancer, was seen in patients with lung metastases with 5-year survival of 13.0% versus 21.9% (*p* = 0.008) in right-sided compared with that of left-sided colon and rectal cancer.

## Discussion

In this study, the incidence of CRCLM was lower than the 50% often cited in the literature, but similar to results from previously reported population-based studies [2, 3]. Liver metastases were more often detected in the lower age groups as compared to the older age groups, which could possibly be attributed to a lower tendency to perform an extensive liver work-up in the old and frail. CRC originating from the left colon and rectum had a higher incidence of liver metastases compared with that of right-sided cancer. In spite of that, patients diagnosed with liver metastases secondary to a right-sided colon cancer had a higher T- and N-stage stage at diagnosis. Previously published retrospective analyses have shown the same pattern, with right-sided colon cancer having a higher TNM stage at diagnosis [11, 16] and lower incidences of liver metastases [6, 10]. Price et al. speculated on whether the delay in diagnosis for right-sided cancer could result in more extensive metastatic disease at diagnosis, explaining the worse survival in metastatic right-sided cancer [7]. The present study supports this hypothesis with a higher number of metastases and more liver segments involved at detection, observed in patients with liver metastases from a right-sided colon cancer. Since most other studies present the presence of metastatic disease as a dichotomised value (yes or no), the information regarding differences in the actual extent of the disease is usually scant. Lung metastases, typically detected a year later than liver metastases, were also significantly more often detected in patients with left-sided colon and rectal cancer, compared to patients with right-sided cancer, while peritoneal dissemination was more often seen in right-sided cancer. There are known molecular differences between right- and left-sided colon cancer with the former more often being poorly differentiated, as well as more often *KRAS* and/or *BRAF* mutated [8, 10, 17]. RAS mutations have been reported to be associated with a more aggressive tumour biology and occur in 35–45% of all patients with metastatic CRC [18, 19]. Among patients with resectable liver metastases, the presence of RAS mutations is 10–15% lower, indicating that the underlying biology is prognostic and influences the likelihood of surgical resection [20]. Furthermore, patients with RAS mutations undergoing liver resection have worse overall and recurrence-free survival [20].

Since this study included reviews of abdominal imaging, detailed information of intrahepatic metastatic spread can be presented. Almost one-third of all patients with metastases in the liver had widespread disease at diagnosis, engaging all segments. On the other hand, nearly 50% had liver-only metastases at presentation with metastatic disease, indicating a more pronounced intrahepatic spread than previously reported [2, 3]. This might in part be explained by the true population-based nature of this study, where patients were not excluded from analysis based on treatment status of the primary tumour.

Patients with liver metastases from a right-sided cancer had a significantly worse overall survival compared to patients with liver-metastasized left-sided colon cancer. This is in agreement with previous reports of inferior survival of right-sided colon cancer in the presence of metastatic disease [7, 14]. Norén et al. also reported better survival in liver metastatic left-sided CRC and further demonstrated that the resection rate of liver metastases varied according to site of the primary tumour, with lower resection rates for right-sided cancers [6]. This was also found to be true in the present study, where patients with liver metastatic right-sided cancer were resected for their liver metastases less often and had an intermediate survival as compared to patients resected for liver metastases from a left-sided cancer and patients not resected at all. However, tumour site was not significantly associated with the likelihood of undergoing a resection in the multivariate logistic regression analysis. Reasons for these survival differences are most likely multifactorial. Patients with right-sided cancer present later, are older at diagnosis and may have more comorbidities than patients with left-sided cancer [11, 21]. In a study by Gervaz et al. the difference in stage could not explain survival differences, since the survival difference was still present when adjusting for multiple factors, including stage [11]. The stage-adjusted subgroup analysis in this study was limited by the low number of patients in each stage group but still, the results were similar to other studies, with stage III and IV right-sided cancer having significantly worse survival compared with that of left-sided cancer [8, 12]. Stage II right-sided cancer on the other hand is associated with better survival compared with that of left-sided [12, 13, 22]. This might be explained by a higher proportion of right-sided stage II cancers having microsatellite instability, which in turn is associated with a more favourable outcome and a decreased likelihood of distant organ metastases at diagnosis [23]. In a recent publication by Warschkow et al., using propensity score analysis on patients with resected stage I-III colon cancer, a worse survival for left-sided colon cancer was found which contradicts the previous paradigm [13]. A Danish population-based study challenged the right/left categorization since no clear trend was found regarding survival and their more detailed colon sub-site analysis revealed a much more complex picture [9].

The proximal and distal colon segments are of different embryological origins, where the former develops from the midgut and the latter from the hindgut. Most other studies on location of colon cancer as a prognostic factor for survival do not include rectal cancer. In the few studies that do, the results are consistent with right-sided cancer having worse survival compared with left-sided cancer [7, 10] When analysing early stage disease, there could be merit in not including rectal cancer in in the analyses due to different treatment strategies, but in the setting of metastatic disease treatment of metastases is not different between metastatic rectal and colon cancer. Even when excluding rectal cancer from left-sided colon cancer in the subgroup analysis in this study, the findings remained comparable with primary tumour site still being a statistically significant prognostic factor, influencing survival.

The location of extra-hepatic disease is known to affect survival, with lung metastases having better outcomes [24]. The present study shows that patients with metastases confined to the lungs did much better and had three times longer median survival (4.3 years) compared to patients with liver-only metastases. In the multivariate analysis of factors influencing survival, the presence of lung metastases did not significantly influence overall survival in patients with liver metastases, nor in the overall patient population. This finding could stimulate the debate on whether there is merit in treating liver metastases in a subset of patients with unresectable lung metastases. Such an approach is also supported by data reported by Dave et al. showing a 30% 5-year survival in a cohort of patients that were planned for liver and lung resection, but where the pulmonary treatment was not carried out due to tumour progression after liver resection [25]. The expected 5-year survival in these patients were much less, indicating that resection of the liver metastases had an influence on survival.

In the present study the referral rate to a liver MDT was higher than the figure reported by Young et al. where 22.5% of patients with CRCLM were referred for evaluation by a liver surgeon [26]. As a likely consequence, the resection rate of liver metastases in this study (25.4%) were higher than the resection rate in the Young study (17%) [26], but in the same range as other population-based studies [3]. The 5-year survival rate of 48.6% in patients resected for liver metastases presented in this study indicates that high survival rates can be achieved even in a population-based setting, where referral and selection bias could be assumed as being minimal. It is clear that with applying current indications for curative-intended treatment of CRCLM, not all resected patients will benefit from the procedure in terms of OS. Conversely, some patients with liver metastases that currently are assessed as not suitable for curative-intended treatment might have a survival benefit from hepatic disease-control.

A weakness of the present study is the retrospective design where follow-up of patients could have been influenced by differences in adherence to guidelines by the treating physicians. Some patients could have had a less stringent follow-up because of advanced age, severe comorbidities or early carcinomas perceived to have a lower risk for metastasizing. Even if it is unlikely to have affected overall survival, it could to some extent have affected the time of detection of metastases. A major limitation of this study is the lack of knowledge of the American Society of Anaesthesiologists (ASA) classification that could potentially be a confounder in the survival analyses. Due to the limited number of patients in the group of metastatic right-sided colon cancer, a proper stage-adjusted analysis could not be conducted. This of course makes inference on survival in these groups uncertain. Lung metastases did not significantly influence survival in the multivariate Cox regression analysis, but one cannot exclude immortality bias, since lung metastases were found to be diagnosed later in the course of the disease. Furthermore, no information on tumour markers and mutation status of the primary tumours was available, which could possibly shed light on the difference in survival seen between liver metastatic right-sided and left-sided colon cancer.

## Conclusion

In conclusion, the study offers detailed population-based data on the metastatic pattern of CRC that could assist in more structured and individualized guidelines for follow-up and treatment of CRCLM. It also supports earlier findings regarding metastatic CRC, namely that right-sided tumours are associated with worse outcomes compared to left-sided tumours. Moreover, data suggest that lung metastases are not always associated with poor survival. With increasing focus on the benefit of liver intervention in terms of prolonged survival, there might be a subgroup of patients that could benefit from liver intervention, despite the presence of lung metastases not suitable for treatment.

## Additional file

Additional file 1: Table S1. The influence of primary tumour location on survival in liver metastatic colorectal cancer, stratified by stage. Cox regression model adjusted for: age, sex, synchronous versus metachronous, size of largest liver metastasis (50), number of liver metastases, liver resection, lung metastases and stratified by stage. (DOCX 12 kb)

## Abbreviations

ASA: American Society of Anaesthesiologists
CI: Confidence interval
CRC: Colorectal cancer
CRCLM: Colorectal cancer liver metastases
FLR: Future liver remnant
HR: Hazard ratio
MDT: Multidisciplinary team
OR: Odds ratios
OS: Overall survival
SCRCR: Swedish National Quality Registry for Colorectal Cancer Treatment
