# Supplementary material for: Colorectal cancer liver metastases – a population-based study on incidence, management and survival
Source: BMC Cancer. 2018 Jan 15;18:78. doi: 10.1186/s12885-017-3925-x (PMC5769309; doi:10.1186/s12885-017-3925-x)
Supplement: Additional file 1: Table S1. — The influence of primary tumour location on survival in liver metastatic colorectal cancer, stratified by stage. Cox regression model adjusted for: age, sex, synchronous versus metachronous, size of largest liver metastasis (50), number of liver metastases, liver resection, lung metastases and stratified by stage. (DOCX 12 kb) [file 12885_2017_3925_MOESM1_ESM.docx]

**Table S1**

|  | All stages | | | Stage II n=25 | | | Stage III n=54 | | | Stage IV n=187 | | |
| --- | --- | --- | --- | --- | --- | --- | --- | --- | --- | --- | --- | --- |
|  | HR | 95%CI | P | HR | 95%CI | P | HR | 95%CI | P | HR | 95%CI | P |
| Unadjusted |  | | |  | | |  | | |  | | |
| Right-sided | 1.00 |  | | 1.00 |  |  | 1.00 |  |  | 1.00 |  |  |
| Left-sided^a^ | 0.48 | 0.36-0.64 | <0.001 | 3.00 | 0.40-22.70 | 0.287 | 0.16 | 0.07-0.37 | <0.001 | 0.61 | 0.45-0.84 | 0.003 |
|  | | | | | | | | | | | | |
| Adjusted for all covariates ^b^ | HR | 95%CI | P | HR | 95%CI | P | HR | 95%CI | P | HR | 95%CI | P |
| Right-sided | 1.00 |  |  | 1.00 |  |  | 1.00 |  |  | 1.00 |  |  |
| Left-sided^a^ | 0.64 | 0.48-0.86 | 0.003 | 6.40 | 0.62-66.00 | 0.119 | 0.13 | 0.05-0.35 | <0.001 | 0.65 | 0.47-0.90 | 0.009 |

^a^ Including rectal cancer. ^b^ Cox regression model adjusted for: Age, sex, synchronous versus metachronous, size of largest liver metastasis (50), number of liver metastases, liver resection, lung metastases. From date of diagnosis of CRC.
